# Hamartome lipomateux superficiel de Hoffmann-Zurhelle

**DOI:** 10.11604/pamj.2015.21.31.4773

**Published:** 2015-05-15

**Authors:** Sanaa Krich, Mariame Meziane, Hanae Bouzidi, Youssef Bouyahyaoui, Salim Gallouj, Amal Bennani, Kaoutar Moumna, Taoufik Harmouch, Aafaf Amarti, Fatima-Zohra Mernissi

**Affiliations:** 1 Service de Dermatologie, CHU Hassan II de Fès, Maroc; 2 Service d'Anatomopathologie CHU Hassan II de Fès, Maroc

**Keywords:** Hamartome lipomateux, Hoffmann-Zurhelle, tumeur bénigne, lipomatous hamartoma, Hoffmann-Zurhelle, benign tumor

## Abstract

L'hamartome lipomateux superficiel de Hoffmann-Zurhelle est une tumeur bénigne souvent congénitale. Histologiquement, il est caractérisé par la présence hétérotopique de cellules adipeuses quelquefois lipoblastiques autour des trajets vasculaires dermiques. Nous rapportons une nouvelle observation de forme multiple à révélation tardive chez une femme âgée de 31 ans sans antécédents pathologiques notables qui a été adressée à la consultation pour des papules et tumeurs asymptomatiques de couleur chaire se regroupent en placards à disposition linéaire et zostèriforme au niveau de la face externe de la cuisse droite depuis l’âge de 13 ans, augmentant progressivement de taille. L’étude histologique d'un fragment biopsique avait montré un épiderme régulier, plicaturé et kératinisant, soulevé par un tissu fibro-adipeux abondant incluant quelques vaisseaux sanguins aux dépens du derme moyen. Ces données cliniques et histologiques ont permis de retenir le diagnostic d'hamartome lipomateux superficiel. Une exérèse chirurgicale des tumeurs de grande taille a été proposée complété par le laser CO2 pour le reste de lésions cutanées. L'hamartome lipomateux superficiel est une lésion bénigne sans potentiel de malignité. L'exérèse chirurgicale peut être proposée si la lésion est gênante ou dans un but essentiellement esthétique.

## Introduction

L'hamartome lipomateux superficiel de Hoffmann-Zurhelle est une tumeur bénigne rare souvent congénitale [1]. Histologiquement, il est caractérisé par la présence hétérotopique de cellules adipeuses quelquefois lipoblastiques autour des trajets vasculaires dermiques [2]. Nous rapportons une nouvelle observation de forme multiple à révélation tardive.

## Patient et observation

**Figure 1:**
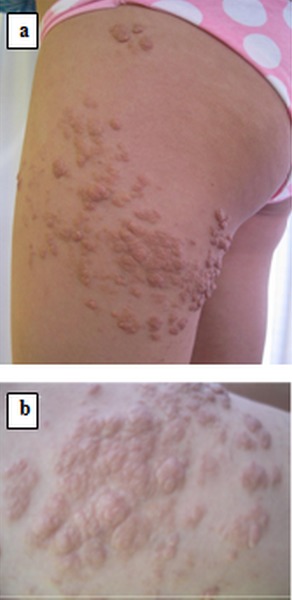
Papules et tumeurs polylobées se regroupent en placards à disposition linéaire et zostèriforme (a, b)

**Figure 2:**
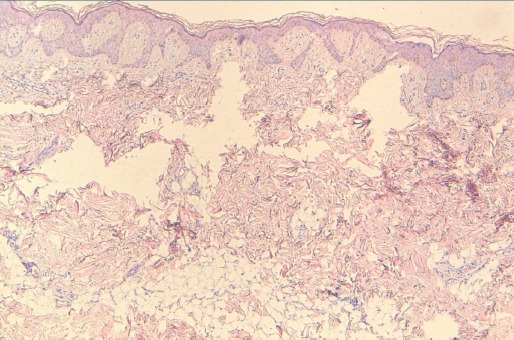
Tissu fibro-adipeux incluant quelques vaisseaux sanguins aux dépens du derme moyen

Une femme âgée de 31 ans sans antécédents pathologiques notables qui a été adressée à la consultation de dermatologie pour des papules et des tumeurs asymptomatiques au niveau de la cuisse droite depuis l’âge de 13 ans, augmentant progressivement de taille. L'examen clinique retrouvait des papules et tumeurs polylobées de consistance molle, de couleur chaire se regroupent en placards à disposition linéaire et zostèriforme au niveau de la face externe de la cuisse gauche (Figure 1). Le reste de l'examen dermatologique et somatique était sans particularité. L’étude histologique d'un fragment biopsique avait montré un épiderme régulier, plicaturé et kératinisant, soulevé par un tissu fibro-adipeux abondant incluant quelques vaisseaux sanguins aux dépens du derme moyen (Figure 2). Ces données cliniques et histologiques permettaient de retenir le diagnostic d'hamartome lipomateux superficiel. Une exérèse chirurgicale des tumeurs de grande taille a été proposée complété par le laser CO2 pour le reste de lésions cutanées.

## Discussion

L'hamartome lipomateux superficiel (ou nævus lipomateux cutané superficiel) est une hétérotopie adipocytaire rare, décrite initialement par Hoffman et Zurhelle en 1921 [1]. On distingue deux types cliniques [2, 3]: la forme multiple, dite classique et la forme solitaire. La forme classique se présente sous l'aspect d'amas de papules ou nodules de couleur chair ou jaunâtres, de consistance molle, siégeant généralement dans la région lombosacrée ou sur les cuisses. Elles peuvent être multifocales, à disposition linéaire ou zostériforme comme le cas chez notre patiente. Ces lésions peuvent être présent à la naissance ou survenir dans les deux premières années de la vie. Mais des formes tardives sont plus rares [4]. Notre malade est caractérisé par la présence d'une forme multiple de révélation tardive. La forme solitaire s'observe à un âge plus tardif et se localise souvent au niveau du cuir chevelu, nez ou clitoris [5]. L'examen histologique permet de confirmer le diagnostique en montrant des amas ectopiques de tissu adipeux dans le derme moyen. Le diagnostic différentiel se pose essentiellement avec l'hypoplasie dermique en aires, les nævus mélanocytaires et le molluscum pendulum.

## Conclusion

L'hamartome lipomateux superficiel est une lésion bénigne sans potentiel de malignité. L'exérèse chirurgicale peut être proposée si la lésion est gênante ou dans un but essentiellement esthétique.
